# Activation of Methanogenesis in Arid Biological Soil Crusts Despite the Presence of Oxygen

**DOI:** 10.1371/journal.pone.0020453

**Published:** 2011-05-31

**Authors:** Roey Angel, Diethart Matthies, Ralf Conrad

**Affiliations:** 1 Max-Planck-Institute for Terrestrial Microbiology, Marburg, Germany; 2 Plant Ecology, Department of Ecology, University of Marburg, Marburg, Germany; Argonne National Laboratory, United States of America

## Abstract

Methanogenesis is traditionally thought to occur only in highly reduced, anoxic environments. Wetland and rice field soils are well known sources for atmospheric methane, while aerated soils are considered sinks. Although methanogens have been detected in low numbers in some aerated, and even in desert soils, it remains unclear whether they are active under natural oxic conditions, such as in biological soil crusts (BSCs) of arid regions. To answer this question we carried out a factorial experiment using microcosms under simulated natural conditions. The BSC on top of an arid soil was incubated under moist conditions in all possible combinations of flooding and drainage, light and dark, air and nitrogen headspace. In the light, oxygen was produced by photosynthesis. Methane production was detected in all microcosms, but rates were much lower when oxygen was present. In addition, the δ^13^C of the methane differed between the oxic/oxygenic and anoxic microcosms. While under anoxic conditions methane was mainly produced from acetate, it was almost entirely produced from H_2_/CO_2_ under oxic/oxygenic conditions. Only two genera of methanogens were identified in the BSC-*Methanosarcina* and *Methanocella*; their abundance and activity in transcribing the *mcrA* gene (coding for methyl-CoM reductase) was higher under anoxic than oxic/oxygenic conditions, respectively. Both methanogens also actively transcribed the oxygen detoxifying gene catalase. Since methanotrophs were not detectable in the BSC, all the methane produced was released into the atmosphere. Our findings point to a formerly unknown participation of desert soils in the global methane cycle.

## Introduction

Methane is the third most important greenhouse gas on Earth after water vapour and CO_2_ [1]. Traditionally, methane is thought to have 25 times the global warming potential (GWP) of CO_2_, but recent models, which take into account direct and indirect interactions with aerosols, estimate its GWP to be as high as 26 to 41 times that of CO_2_ over a 100-year horizon [2]. Of the 500–600 Tg CH_4_ emitted annually into the atmosphere about 74% is biogenic, i.e. the product of methanogenesis [1]. Despite the biogeochemical importance of methanogenesis as a terminal electron sink in anoxic environments, only one group of microorganisms, the methanogenic Archaea (methanogens), are able to produce methane. The methanogens themselves are phylogenetically divided into 5 families within the phylum Euryarchaea and are comprised of 31 known genera. Biogenic methane can be produced from a wide range of methylated compounds, but in most natural systems methane arises from two pathways only: reduction of CO_2_ (hydrogenotrophic methanogenesis) and cleavage of acetate (acetoclastic methanogenesis) [3]. One exception is saline and hypersaline environments such as marine sediments and salt lakes where methanogenesis from methylated compounds such as trimethylamine can play a significant role [4].

The traditional textbook notion is that methanogenesis occurs only in highly reduced, anoxic environments such as wetlands, rice fields, lentic and marine sediments as well as in rumens and in the guts of termites. This notion is based on two aspects of the physiology of methanogens: 1) they are strict anaerobes and the presence of oxygen leads to the formation of reactive oxygen species (ROS), which damage cell membranes, DNA and proteins [5]. Particularly in methanogens, oxygen causes an irreversible dissociation of the F420-hydrogenase enzyme complex, a crucial electron transporter in methanogenesis [6]. Indeed, methane production in an active rice paddy soil was shown to cease completely upon oxygen stress [7]. 2) Methanogens are poor competitors for hydrogen and acetate with nitrate, iron and sulphate reducers. Thus, even in the absence of oxygen, hydrogenotrophic or acetoclastic methanogenesis only commences once most nitrate, ferric iron and sulphate in the system are depleted [8]. Nevertheless, it has been previously shown that many soils which are typically aerated, including a desert soil, can turn methanogenic when incubated under anoxic conditions as slurry [9].

Deserts (semiarid, arid and hyperarid regions) span over 44 mil. km^2^ and make up 33% of the Earth's land surface [10]. Desert soils are typically covered by a unique crust, of a few millimetres, densely colonized by microorganisms. These include primarily polysaccharide-secreting/photosynthetic microorganisms such as cyanobacteria and microalgae, but also fungi, lichens and mosses, as well as an array of prokaryotic species about which little is known [11]. These biological soil crusts (BSCs) are mostly inactive when dry but regain nearly full photosynthetic activity within hours to a few days upon wetting [12]. As a result of their high microbial activity and of their compact structure, oxygen becomes limiting very quickly in active BSCs and anoxic microniches are formed within it, where anaerobic respiration and eventually fermentation processes can potentially take place [13]. If true, this would not be the first case where photosynthetic microorganisms and anaerobes co-occur in nature. Microbial mats in which oxygenic cyanobacteria and anaerobes (including methanogens) live in close proximity are common in marine and hypersaline environments and represent one of the most ancient life forms on Earth. This life form probably originated in the Mid-Late Archaean (ca. 3.5 Ga ago) and was the dominant photosynthetic system on Earth prior to the emergence of plants [14]. We hypothesized that although they are strict anaerobes, some methanogens are able to endure long periods of exposure to oxygen in the BSC when it is dry and take advantage of anoxic micro-niches and fresh organic matter which are formed after a rain event.

We used microcosms and simulated different natural conditions following a rain event to investigate a possible methanogenic activity in BSCs, in particular when exposed to atmospheric oxygen levels. A three-factorial experiment was set up varying 3 sets of factors: flooded/wet-drained, oxic/anoxic, and light/dark, in all possible combinations (**Figure S1**).

## Results and Discussion

### Methanogenesis under oxic atmosphere

**Figure 1:**
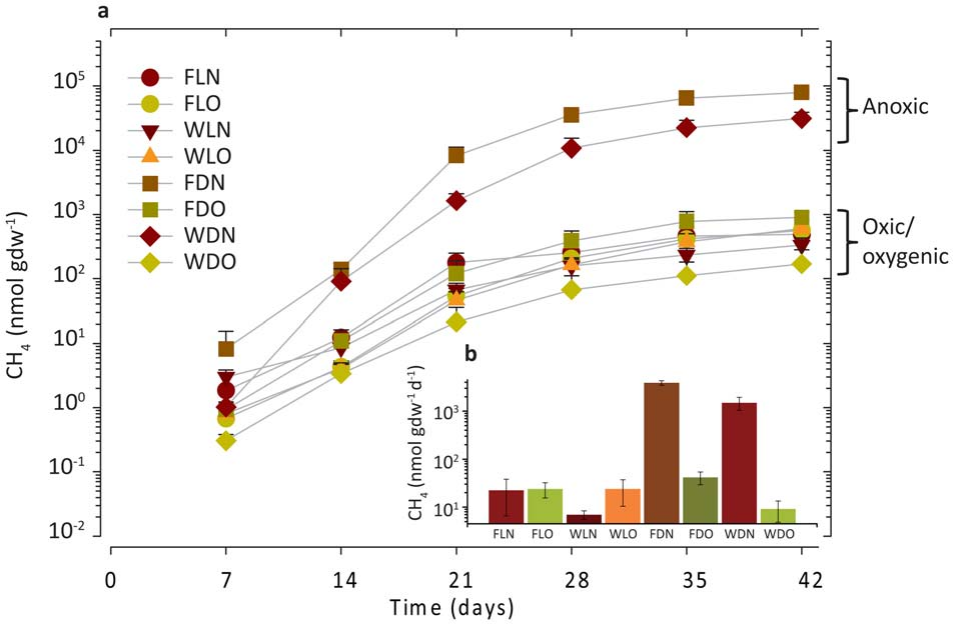
Methane production in the microcosms throughout the incubation. A. Accumulation of CH_4_ in the microcosm headspaces B. mean production rate per day: means±1 SE; n = 3. Treatment codes are as follows: flooded-F, wet-drained-W, light-L, dark-d, N_2_ headspace-N, air (21% O_2_) headspace-O.

Methane was detectable in the headspace of all microcosms seven days after the start of the experiment and it continuously accumulated throughout the incubation, regardless of treatment (**Figure 1A**). The lag in the methane detection can be due to the time it takes for oxygen, and potentially other alternative electron acceptors, to be depleted and/or to the recovery and growth of the methanogenic population. A strong, two orders of magnitude, difference in the methanogenic activity was seen between the oxic and the anoxic microcosms incubated in the dark. These anoxic treatments-FDN and WDN-accumulated methane at a rate of 3800±400 and 1500±400 nmol gdw^−1^ d^−1^, respectively, while the parallel oxic treatments-FDO and WDO-accumulated methane at a rate of 41.6±12.4 and 9.2±4.3 nmol gdw^−1^ d^−1^, respectively (**Figure 1B**, **Table S1**). The microcosms incubated in the light showed similar methane production rates to the dark oxic microcosms (21.7±3.7 nmol gdw^−1^ d^−1^ on average), and indeed methane production rates between these treatments were not significantly different, indicating no apparent effect of initial oxygen levels (P = 0.66 in a t-test).

Active production of oxygen due to photosynthesis was observed in the microcosms incubated in the light (**Figure S2A**). The oxygen fluxes modelled from vertical oxygen concentration profiles ranged from 10–20 nmol cm^−2^ s^−1^ (**Figure S3**). Oxygen in these microcosms penetrated down to about 1500 µm, i.e. half the depth of the crust, below which the soil was anoxic. In the two oxic microcosms incubated in the dark, oxygen penetrated somewhat deeper down to about 2–2.5 mm. The greater penetration of oxygen into the soil in the dark incubations seems counterintuitive, yet it can be explained by increased microbial activity below the photosynthetic later resulting from the release of labile organic compounds by the primary producers. Overall, oxygen penetration depth was in agreement with other measurements performed on wet BSCs [13]. Most of the CO_2_ released from the BSCs accumulated in the microcosm headspaces within the first week, but was much lower in the light treatment where it was most likely used for photosynthesis (**Figure S2B**; F_1,16_ = 107.6, P<0.01). Hydrogen levels were, however, higher in the light than in the dark treatments (**Figure S2C**; F_1,16_ = 5.4, P = 0.03). In fact, hydrogen in the oxic dark treatment was below the detection limit throughout the experiment (<2.5 Pa).

Both light and oxygen treatments strongly reduced methane production while flooding increased it (**Figure 1B** **; Table S1**). The effects of light and oxygen interacted strongly, reflecting the fact that the effect of oxygen treatment on methane production was dependent on light (as the latter promoted photosynthesis in the BSCs). Methane production rates were strongly negatively correlated with the depth of the anoxic boundary (less than 1% O_2_), but only weakly with water content (**Table S2**). This strong correlation demonstrates the well known negative effect of oxygen on the methanogenic process, which was the primary factor affecting methanogenesis in our experiment.

### Phylogenetic analysis of the *mcrA* gene revealed only two active methanogenic types

**Figure 2:**
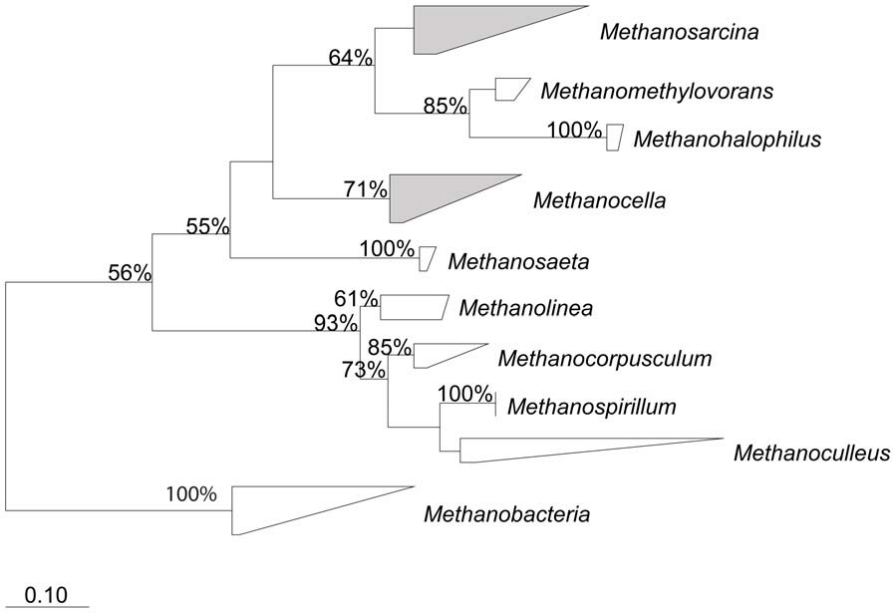
Maximum likelihood phylogenetic tree based on aligned partial amino acid sequences of the methyl coenzyme M reductase gene (*mcrA*). Amino acid composition was deduced from DNA sequences and the tree was calculated with RAxML 7.04. Bootstrap values above 50% (out of a 100 trials) are displayed next to the nodes. Shaded clusters with diagonal lines contain sequences that were detected in the soil samples.

In contrast to other methanogenic environments, which typically host many methanogenic species simultaneously [15]–[17], the diversity in our microcosms was remarkably low. Analysis of the *mcrA* gene sequences revealed only two very tight clusters of sequences closely related to either *Methanosarcina*, which produces methane from a variety of substrates including acetate and H_2_/CO_2_ [18], or *Methanocella*, which is capable of hydrogenotrophic methanogenesis only [19](**Figure 2**).

**Figure 3:**
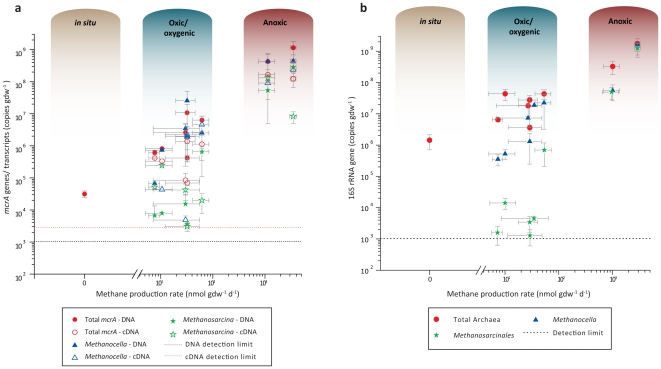
Gene and transcript copy numbers quantified using qPCR plotted against methane production rates: means±1 SE; n = 3. In situ refers to the dry BSC prior to any treatment. A. *mcrA* gene and transcript copy numbers. B. 16S rRNA gene copy numbers.

Thanks to this low-complexity methanogenic community we could individually quantify the 16S rRNA gene copies and *mcrA* gene and transcripts copies for the two methanogenic types as well as generally quantify the 16S rRNA gene of the archaeal community, and the total *mcrA* gene and transcript copies. We observed differences between individual treatment combinations, but by far the strongest effects were a smaller methanogenic community and lower transcription levels in the oxic/oxygenic compared to the anoxic microcosms (**Figure 3**). 16S rRNA and *mcrA* gene copies were in the range of 10^8^–10^9^ copies gdw^−1^ in the anoxic but only 10^3^–10^7^ in the oxic/oxygenic microcosms. In all treatments we observed an increase in the quantity of *mcrA* gene copies from 3.15×10^4^ copies gdw^−1^ in the soil before incubation to at least 4.16×10^5^ copies gdw^−1^ (a tenfold increase in the WLO treatment) and up to 1.15×10^9^ copies gdw^−1^ in the FDN treatment (an increase of almost five orders of magnitude). Apart from a general effect of oxygen on the community size and gene expression, we noted a differential effect on *Methanocella* and *Methanosarcina*. The ratio of *Methanosarcina* to total 16S rRNA gene copies was significantly lower in the oxic/oxygenic than in the anoxic treatments (**Table S1**, **Figure 3**). In contrast to the effect on methane production, flooding did not have a significant effect on the ratio of *Methanosarcina* to total 16S rRNA gene copies. The same trend was seen for the ratio of *Methanosarcina mcrA* to the general *mcrA* gene and transcript copies, while no such effects could be seen for the ratio of *Methanocella* to total 16S rRNA gene and *mcrA* gene and transcript copies (All tests P>0.24).

### Stable isotope analysis revealed different methanogenic pathways under oxic and anoxic headspaces

**Figure 4:**
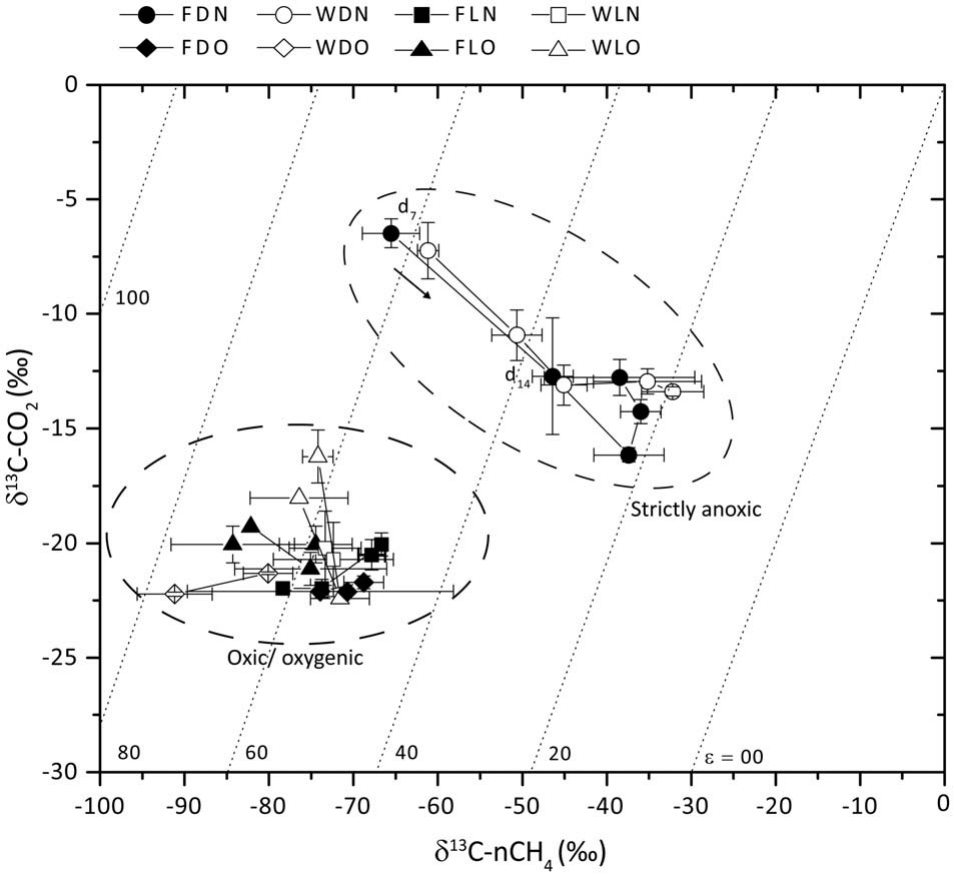
Stable carbon isotope signature (δ^13^C) of the CO_2_ and the newly formed methane in the microcosm headspaces (see Methods): means±1 SE; n = 3. Isolines represent different apparent fractionation factors (ε_app_; eq.3). “Strictly anoxic” refers only to the anoxic microcosms in the dark while “oxic/oxygenic” refers to all the rest. The arrow in the “strictly anoxic” ellipse points to the direction of temporal development (d7 and d14 Refer to day 7 and 14th resp.). Treatment codes are as follows: flooded-F, wet-drained-W, light-L, dark-D, N_2_ headspace-N, air (21% O2) headspace-O.

We also analyzed the stable isotope signature of the carbon in methane and CO_2_ (^13^C:^12^C) to decipher the proportional contribution of different methanogenic pathways [3]. Our analysis of isotopic signatures (**Figure 4**) revealed two distinct clusters: the strictly anoxic microcosms had δ^13^C-nCH_4_ (isotopic signature of the newly formed methane) average values of −63‰ in the first week of incubation, an average of −35‰ throughout the rest of the incubation period, and δ^13^C-CO_2_ values of −16 to −7‰. The oxic/oxygenic microcosms showed lighter isotopic signatures with average δ^13^C values of −75‰ and −20‰ for methane and CO_2_, respectively, which were stable over time. The difference between the isotopic signatures of the CO_2_ in the two clusters (**Figure 4**) can be related to the difference in the signature of the organic carbon and the carbonate reservoir in the soil, which constituted up to 34% of the soil mass [20]. The δ^13^C of carbonate (−4.09‰) was heavier than that of organic carbon (−20.5‰). In the oxic/oxygenic microcosms CO_2_ was probably produced only from organic matter. However, in the anoxic microcosms, the CO_2_ was probably also generated from the carbonate. The contribution of carbonate may be attributed to the release of CO_2_ from the reaction of the calcium carbonate in the soil with acids, which are associated with anaerobic degradation processes (**Table S3**).

The δ^13^C values of CO_2_ and CH_4_ allow the calculation of average apparent fractionation factors (α_app_; eq.2). The α_app_ for the strictly anoxic microcosms was 1.025±0.002, which was much smaller than the α_app_ = 1.065±0.003 obtained for the oxic/oxygenic microcosms, indicating different methanogenic pathways under the two treatments. The large fractionation factor obtained for the oxic/oxygenic microcosms is well within the range of 1.040–1.080 typically seen for hydrogenotrophic methanogenesis in pure cultures and in soils at moderate temperatures [3]. Indeed, also for these samples we obtained a fractionation factor of 1.066 when acetoclastic methanogenesis was inhibited using CH_3_F. We therefore conclude that in oxic/oxygenic microcosms CH_4_ was entirely produced from H_2_/CO_2_. The nearly complete lack of acetoclastic methanogenesis in the oxic/oxygenic microcosms could be the result of competition with heterotrophs that oxidize acetate aerobically. Indeed, acetate concentrations were generally much lower in the pore water of oxic/oxygenic than anoxic microcosms (and so were other fermentation products; **Table S3**).

The fractionation factor of 1.025, obtained in the strictly anoxic microcosms, is similar to that of purely acetoclastic methanogenesis [21], [22]. Therefore, we conclude that acetate contributed substantially to CH_4_ production in these microcosms. To determine more precisely the specific contribution of acetoclastic and hydrogenotrophic methanogenesis to the total methane production in anoxic microcosms, we made the following reasonable assumptions. We assumed that the isotopic signature of the methane in the oxic/oxygenic microcosms was characteristic for hydrogenotrophically produced methane. We further estimated the δ^13^-C of acetoclastically produced methane from the δ^13^-C the soil organic carbon, assuming that the fractionation of organic C to acetate was only small, and that of acetate to methane was either zero or −25.6‰ [23]. By using equation 4 we could then confine the fraction of acetoclastic methanogenesis in the anoxic microcosms to 0.72–1.00 following the first week of incubation.

### The BSC lacks methane oxidizing bacteria

Our comparison of methane production rates and isotopic signatures is based on the assumption that all methane which had been produced was released into the headspace and none of it was oxidized by methane-oxidizing bacteria in the soil, which could potentially reduce the measured concentrations and alter the isotopic signature [24]. We previously showed that active methanotrophs appear to reside only below the BSC, down to a depth of approx. 20 cm [25]. No methane uptake activity and no transcription of the key enzyme in aerobic methane oxidation-the particulate methane monooxygenase (*pmmo*) -could be detected in the BSC itself. We have confirmed this observation also in this study as no *pmmo* transcripts could be detected in the microcosm samples by PCR.

### BSC methanogens transcribe oxygen detoxification genes

For methanogens to be active in a system such as the BSC, which is exposed to atmospheric levels of oxygen throughout most of the year, when dry, and to a constant flux of oxygen, albeit at sub-atmospheric levels, when wet and active, they need to be able to efficiently detoxify reactive oxygen species (ROS). Indeed, it has been previously noted that both *Methanosaricna* and *Methanocella* contain several genes encoding enzymes that detoxify reactive oxygen species. These include enzymes such as catalase (*kat*), superoxide dismutase (*sod*), superoxide reductase (*sor*) and others [26]. The metgenome sequence of RC-I strain MRE50 (now *Methanocella arvoryzae*) contained 7 different putative genes whose function is associated with detoxification of ROS [26]. Since *Methanosarcina* only contains 6 such genes, *Methanocella* is potentially the most oxygen-tolerant methanogen. We tested for the presence of catalase E (*KatE*) gene transcripts using katMsI and katRCI primer pairs for *Methanosarcina* and *Methanocella*, respectively (**Table S4**), and performed phylogenetic analysis. *KatE* transcripts were detected in all treatments and their sequences clustered tightly to their respective methanogen cultivars from which the primers were designed (**Figure S4**). Indeed, the *KatE* sequences retrieved from our microcosms showed a remarkable similarity to those of the cultivated methanogens with only a 1.8% and 4.7% difference in the amino acid sequence for *Methanocella* and *Methanosarcina,* respectively. By comparison, there was a 7.4% and 6.3% difference, respectively, in the *mcrA* sequences at the amino acid level. We compared also the relative expression (transcripts to genes) in differently treated microcosms with respect to the oxygen treatment using qPCR (**Table 1**). Our results show a tendency to an upregulation of *katE* in the oxic vs. the anoxic treatment of each matched pair, yet the standard errors associated with our measurements were in most cases too high to safely conclude that upregulation in response to oxygen is indeed occurring. This is in agreement with the results by Zhang and colleagues (2006) who reported no up regulation of catalase in *Methanosarcina barkeri* in response to air exposure [27], but in contrast to those of Brioukhanov and colleagues [28] who reported the opposite in response to oxidative stress.

**Table 1:** Differences in relative expression (2^−ΔΔCT^)* of *katE* in *Methanocella* and *Methanosarcina* between paired treatments.

Treatment comparison	*Methanocella* †	*Methanosarcina*
FLN-FLO	0.16±0.08	1.36±1.36
WLN-WLO	0.30±0.91	1.09±0.61
FDN-FDO	3.21±0.85	0.35±0.86
WDN-WDO	2.37±1.46	0.06±0.03

Means±1 SE.

*Mean fold change in gene expression.

†Values above 1 represent upregulation in the second matched treatment compared to the first. Treatment codes are as follows: flooded-F, wet-drained-W, light-L, dark-D, N2 headspace-N, air (21% O_2_) headspace-O.

### Ecological relevance

The results presented demonstrate that biological soil crusts which cover the surfaces of deserts around the world are inhabited by methanogens and produce biogenic methane when wet. While methanogens are strict anaerobes, at least some of them are more resilient than so far assumed. Former studies have demonstrated the ability of certain methanogenic cultures to endure desiccation and exposure to high levels of oxygen, probably in resting forms [29], [30]. Here we showed that *Methanosarcina* and *Methanocella* species, in particular, are not only able to tolerate long periods of desiccation in an arid soil, but become metabolically active and start growing within just a few days after wetting even in the presence of oxygen.

It was previously shown that *Methanocella* are usually the most abundant and active methanogens in rice fields [31], [32]. It appears that they are also the dominant methanogens in BSCs. *Methanocella* and *Methanosarcina* spp. have apparently different ecological roles in nature. Although both are cytochrome-containing methanogens, they differ in their substrate range, affinity to hydrogen and growth yield [33]. Our experiments showed differential activity and growth of the two methanogens under different conditions and it is possible that niche differentiation permits their coexistence in soil.

The production of biogenic methane in a BSC proves not just the activity of methanogens but also indicates the activity of a whole community of anaerobes, which constitute a formerly unrecognized part of the BSC biome. These include primary and secondary fermenters, syntrophs and maybe acetogens whose identity in these systems is yet to be elucidated, but which are required for the different stages of the anaerobic degradation cascade [34], [35]. This array of microbes remains inactive during long periods when the soil is dry and saturated with oxygen, but is apparently able to react quickly and take advantage of short periods when water is available and anoxic microniches can be formed.

Furthermore, some hydrogen might be directly transferred from cyanobacteria to the methanogens and used as substrate for methanogenesis as occurs in some hypersaline mats [36]. It is likely that plant litter is part of the organic substrate but primary producing microorganisms such as cyanobacteria probably also provide organic substrate by releasing fresh organic exudates into the soil even when water availability is very low [37], [38]. While cyanobacteria have been shown to be activated by as little as 0.2 mm of rain or even fog or dew [39], it is currently not known what amount of water is required to activate the anaerobic part of the BSC. Assuming the BSC is wet for 2 to 7 weeks a year [40] and using the rates obtained from the oxic/oxygenic microcosms the magnitude of this methane source is estimated at 26 to 92 mg CH_4_ m^−2^ yr^−1^ which amounts to a contribution of 1–4 Tg yr^−1^ from all deserts combined.

Our findings show that BSCs comprise both an aerobic-photosynthetic and an anaerobic-methanogenic part which are simultaneously active. As such, BSCs are widespread terrestrial representatives of the first oxygenic photosynthetic system to emerge on Earth. Their methanogenic activity sheds light on a new and unexpected ecological function of arid soils and might point to a previously unknown contribution of biological soil crusts, and perhaps other aerated soils, to the global methane cycle.

## Materials and Methods

### Soil sampling and characterization

In April 2009 the top 3–4 millimetres of the soil comprising the biological soil crust at an arid site located in the northern Negev Desert in Israel were sampled. The soil is a calcareous silty loam and was previously characterized [25].

### Microcosm design and incubation conditions

Microcosms were designed after Murase and Frenzel [41] with few modifications. In principle, the microcosms were gas-tight PVC and Plexiglas vessels, which consisted of a lower compartment (approximately 60 ml) and an upper compartment (approximately 100 ml) separated by a 0.2 µm hydrophilic polyamide membrane (Whatman). Non-sieved, homogenized fractures of BSC (20 g, each approx. 3 mm ø on average) were placed on top of the membrane and amended with sterile deionized water, thus generating a wet soil layer of approximately 3 mm, mimicking its actual thickness in the field. The bottom compartment of the microcosm contained either sterile deionized water (“flooded” treatment) or 0.1–0.3 mm quartz sand, baked (180°C, 24 h), saturated with sterile deionized water and then drained (“wet-drained”). The upper compartment served as gas headspace, which was flushed with either N_2_ (“anoxic”) or 80% N_2_, 21% O_2_ (“oxic”). Oxygen was supplemented daily to maintain atmospheric levels (“oxic”) or was flushed several times during the incubation with N_2_ to maintain levels below 5% O_2_ (“anoxic” under light). Three replicate microcosms of each of the four possible combinations of treatments were incubated at 25°C in full darkness (“dark”) or under constant light (3000 Lux; “light”) for 42 days (**Figure S1**).

### Gas measurements

For measuring O_2_, H_2_, CO_2_ and CH_4_ gas samples were taken from the headspace of the microcosms at regular time intervals using a gas-tight pressure-lok® syringe (Vici) and analyzed immediately using a gas chromatograph. Methane production rates (nmol gdw^−1^ d^−1^) were calculated for the entire incubation period using linear regression.

### Stable isotopes analysis

The carbon isotope signatures (δ^13^C) of the methane and CO_2_ were determined using GC-C-IRMS against the V-PDB standard as described previously [42]. δ^13^C in the organic matter was analyzed using an elemental analyzer coupled to a mass spectrometer. Measurements were done before and after acidification, the difference being due to carbonate (Nüsslein et al., 2003). Isotopic calculations and estimation of the approximate fraction of hydrogenotrophic methanogenesis of the total methanogenesis were done after Conrad [3]. Briefly, the signature of the newly formed methane between two time points is given by: 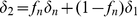
(1)where δ1, δ2 and δn are the isotopic signatures of the methane at times 1 and 2 and of the newly formed, respectively, while *f*n is the fraction of the newly formed methane at time 2.

The apparent fractionation factor for the conversion of CO_2_ to CH_4_ is given by:
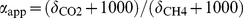
(2)where δ_CO2_ and δ_CH4_ are the isotopic signatures of the carbon in CO_2_ and CH_4_, respectively.

For convenience, ε is often used instead of α. The two can be easily converted through:
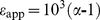
(3)


The relative fraction of H_2_/CO_2_-derived CH_4_ in the total generated CH_4_ was determined from
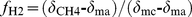
(4)where δ_ma_ and δ_mc_ are the specific isotopic signatures of the carbon in methane produced solely from acetate and H_2_/CO_2_, respectively. To determine δ_mc_, 5 g of BSC were incubated with sterile deionized water (1∶1) in a glass tube and supplemented with 3% CH_3_F which gave complete inhibition of acetoclasitc methanogenesis [43]. The carbon isotope signature was determined during 42 days of incubation as described above.

### Soil samples, pore water analysis and oxygen profiles

After incubations were completed, microcosms were opened and approximately 1 g of soil was sampled for nucleic acid extraction, immediately frozen in liquid nitrogen and stored at −80°C until analysis. Additionally, approximately 2 ml of pore water were collected and analyzed using high pressure liquid chromatography. Water content was determined gravimetrically and oxygen profiles in the soil were determined using an OX-50 glass microsensor (Unisense). The oxygen fluxes were modelled using the Profile V1.0 tool [44]. The anoxic boundary was determined as the depth bellow which oxygen concentration was below 1%.

### Molecular characterization and quantification of archaeal 16S rRNA, *mcrA* and catalase genes

Total nucleic acids were extracted by disrupting 0.5 g of soil in a FastPrep®-24 bead beater in the presence of phosphate buffer, 10% SDS solution and phenol. Following phenol/chloroform purification a subsample was treated with DNase, and the RNA was purified. Random hexamers (0.5 µg) were used for complete cDNA synthesis which was used for amplifying the 16S rRNA and catalase (*KatE*) genes, while for amplification of the methyl coenzyme reductase M gene (*mcrA*) 2 pmol of the mcrA-rev primer were used for *mcrA* cDNA synthesis. All molecular characterizations were done using the primers listed in **Table S4**. Phylogenetic characterization of the methanogenic community was performed by amplifying and cloning the *mcrA* and catalase (*KatE*) genes. Gene and transcript quantifications were done via qPCR (iCycler; Bio-Rad) using either SYBR^®^ Green or dual labelled probe technology. For more details see supplementary information Materials and Methods S1.

### Phylogenetic analysis

Phylogenetic analysis was based on aligned partial amino acid sequences of *mcrA* or *katE*. Amino acid composition was deduced from DNA sequences and the tree was calculated with RAxML 7.04 using rapid hill climbing algorithm and PROTMIX-JTT evolutionary model [45]. Sequences can be retrieved from GenBank® (http://www.ncbi.nlm.nih.gov/genbank/) under accession numbers: HQ269296-HQ269341 and HQ413651-HQ413677 (*mcrA* and *katE* sequences, resp.).

### Statistical analysis

The effects of the three treatments on methane production rates, 16S rRNA and *mcrA* gene copy ratios were analyzed by three-factorial analysis of variance using MATLAB (http://www.mathworks.com). Methane, CO_2_ and H_2_ production rates and gene copy numbers obtained by QPCR were log transformed prior to analysis.

## Supporting Information

Figure S1 **Microcosm incubation conditions used in the experiment.** The bottom compartment of each microcosm contained either water or drained wet sand. Biological soil crust samples were placed on top of a hydrophilic membrane allowing a flow of nutrients and water but not of cells. The headspaces were flushed with either N_2_ or synthetic air (21% O_2_/ 79% N_2_). Microcosms were incubated either in the dark or under full light, in all possible combinations, in triplicates for 42 days.
(TIF)

Figure S2 **Evolution of: a. O_2_, b. CO_2_, c. H_2_ in the microcosm headspaces during the incubation period: means±1 SE; n = 3.** Treatment codes are as follows: flooded-F, wet-drained-W, light-L, dark-D, N_2_ atm. -N, 21% O_2_ atm. -O.
(TIF)

Figure S3 **Vertical soil oxygen profiles in the microcosms.** Only oxic and oxygen producing treatments are shown. Black triangles represent concentration measurements: means±1 SE; n = 3. Blue lines represent O_2_ production zones modelled using Profile V1.0^11^. Treatment codes are as follows: flooded-F, wet-drained-W, light-L, dark-D, N_2_ atm. -N, 21% O_2_ atm. -O.
(TIF)

Figure S4 **Maximum likelihood phylogenetic tree based on aligned partial amino acid sequences of the catalase E gene (** ***katE*** **).** Sequences were obtained using katRCI and katMsr primer pairs targeting the *katE* of *Methanocella* and *Methanosarcina,* respectively. Amino acid composition was deduced from DNA sequences and aligned against an ARB database of catalase sequences. The tree was calculated with RAxML 7.04 using rapid hill climbing algorithm and PROTMIX-JTT evolutionary model. Bootstrap values above 50% (out of a 100 trials) are displayed next to the nodes.
(TIF)

Materials and Methods S1 (DOC)

Table S1 **ANOVA analyses (least squares) testing the effect of the various incubation conditions on methane production rates (nmol d^−1^ gdw^−1^; days 14–42) and ratios of gene and transcript copies.**
(DOC)

Table S2 Pearson correlation coefficients (r) between various measured variables^*^.
(DOC)

Table S3 **Major fermentation products (µM) in the pore water of the microcosms.**
(DOC)

Table S4 **Primers and Probes used in this study.**
(DOC)
