# Supplementary figures and images for: Activation of Methanogenesis in Arid Biological Soil Crusts Despite the Presence of Oxygen
Source: PLoS One. 2011 May 31;6(5):e20453. doi: 10.1371/journal.pone.0020453 (PMC3105065; doi:10.1371/journal.pone.0020453)

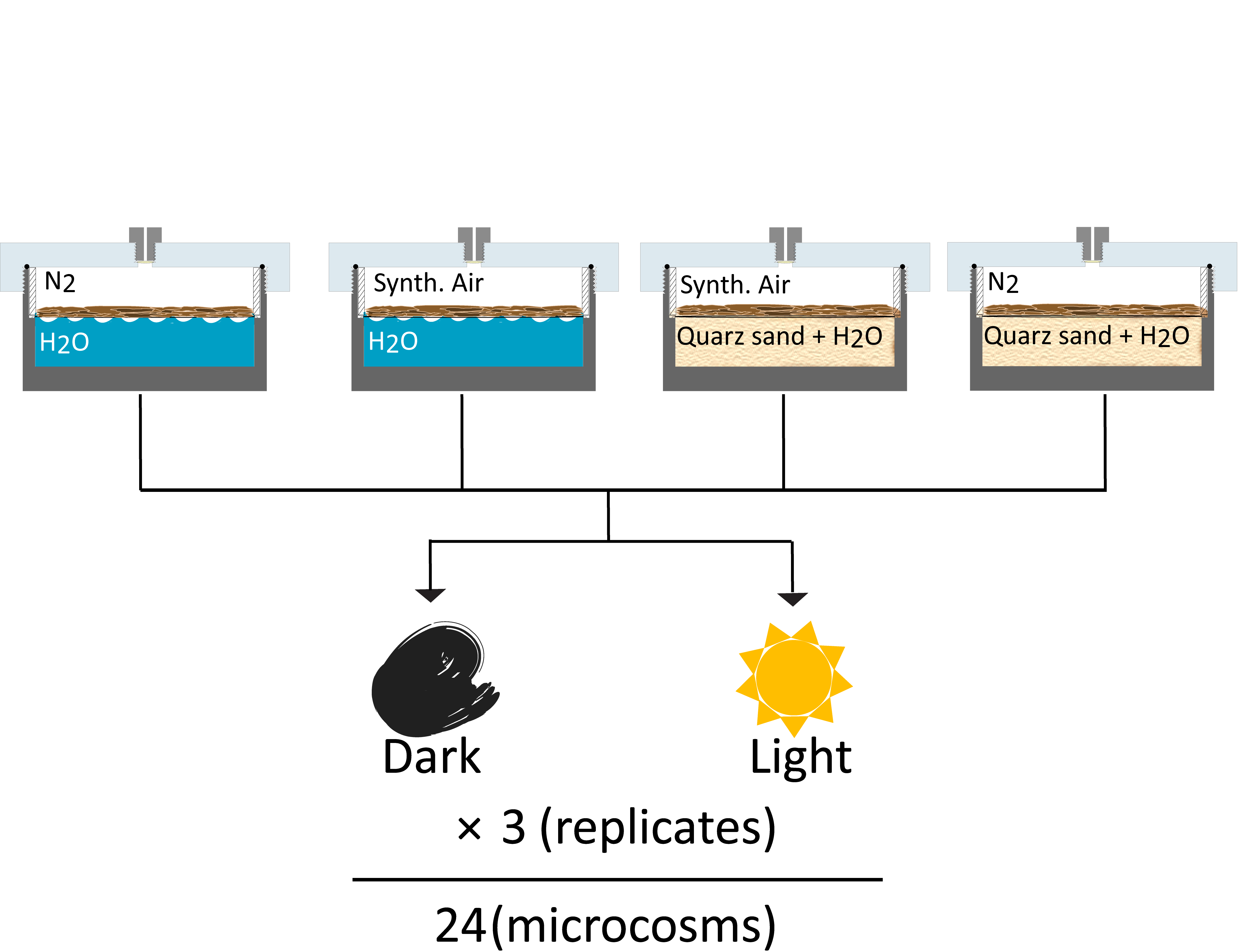

Supplement: Figure S1 — Microcosm incubation conditions used in the experiment. The bottom compartment of each microcosm contained either water or drained wet sand. Biological soil crust samples were placed on top of a hydrophilic membrane allowing a flow of nutrients and water but not of cells. The headspaces were flushed with either N2 or synthetic air (21% O2/ 79% N2). Microcosms were incubated either in the dark or under full light, in all possible combinations, in triplicates for 42 days. (TIF) [file pone.0020453.s001.tif]

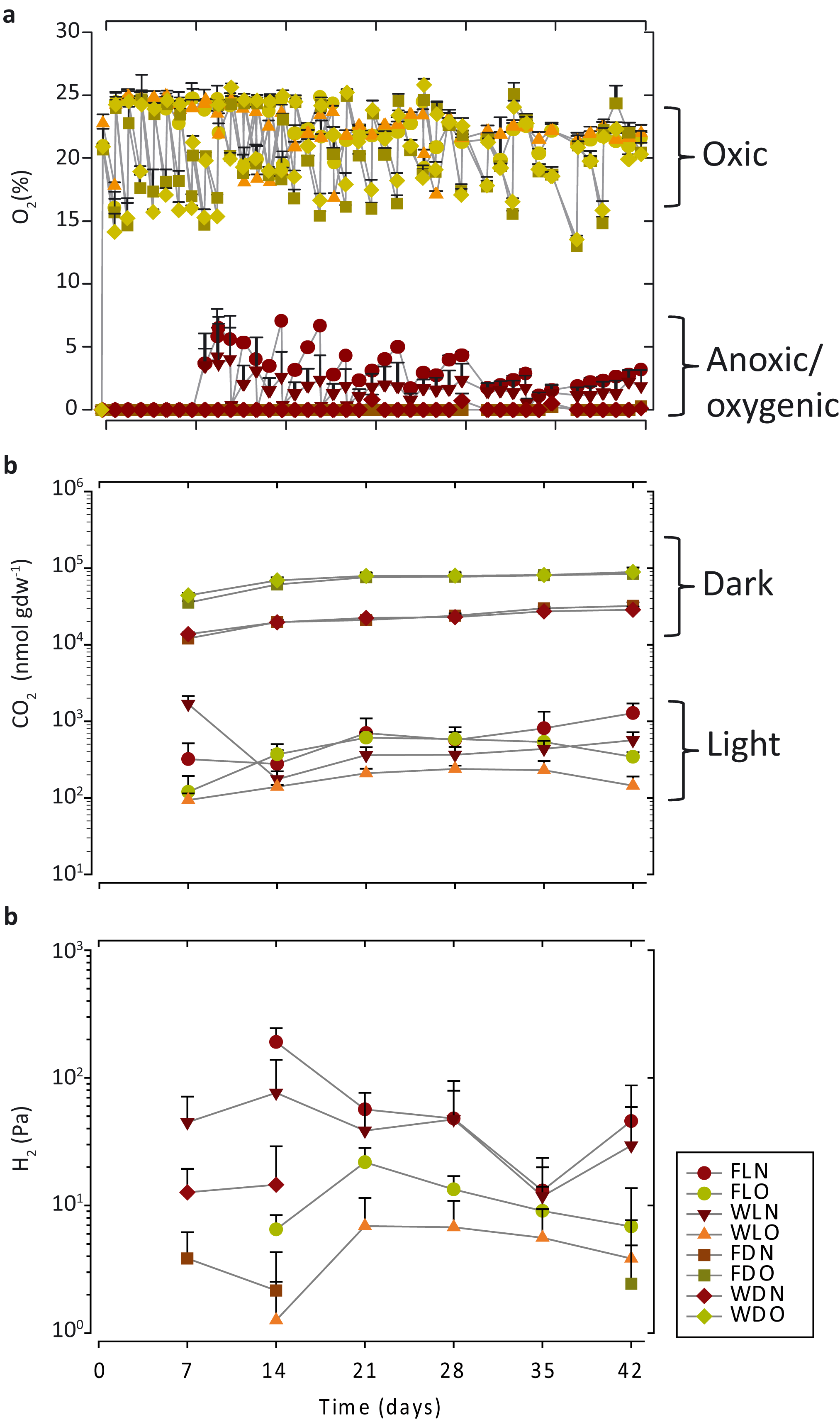

Supplement: Figure S2 — Evolution of: a. O2, b. CO2, c. H2 in the microcosm headspaces during the incubation period: means±1 SE; n = 3. Treatment codes are as follows: flooded-F, wet-drained-W, light-L, dark-D, N2 atm. -N, 21% O2 atm. -O. (TIF) [file pone.0020453.s002.tif]

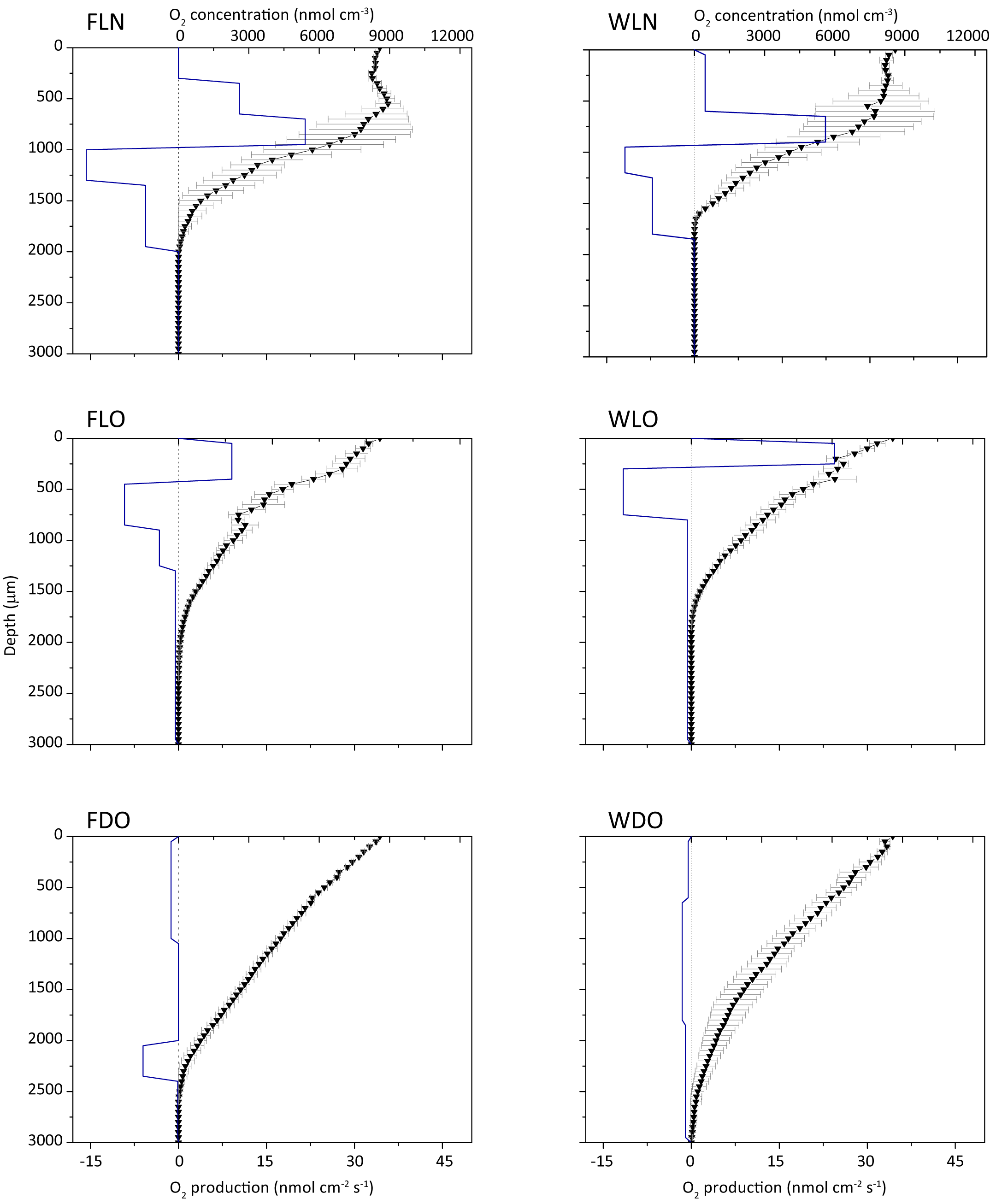

Supplement: Figure S3 — Vertical soil oxygen profiles in the microcosms. Only oxic and oxygen producing treatments are shown. Black triangles represent concentration measurements: means±1 SE; n = 3. Blue lines represent O2 production zones modelled using Profile V1.011. Treatment codes are as follows: flooded-F, wet-drained-W, light-L, dark-D, N2 atm. -N, 21% O2 atm. -O. (TIF) [file pone.0020453.s003.tif]

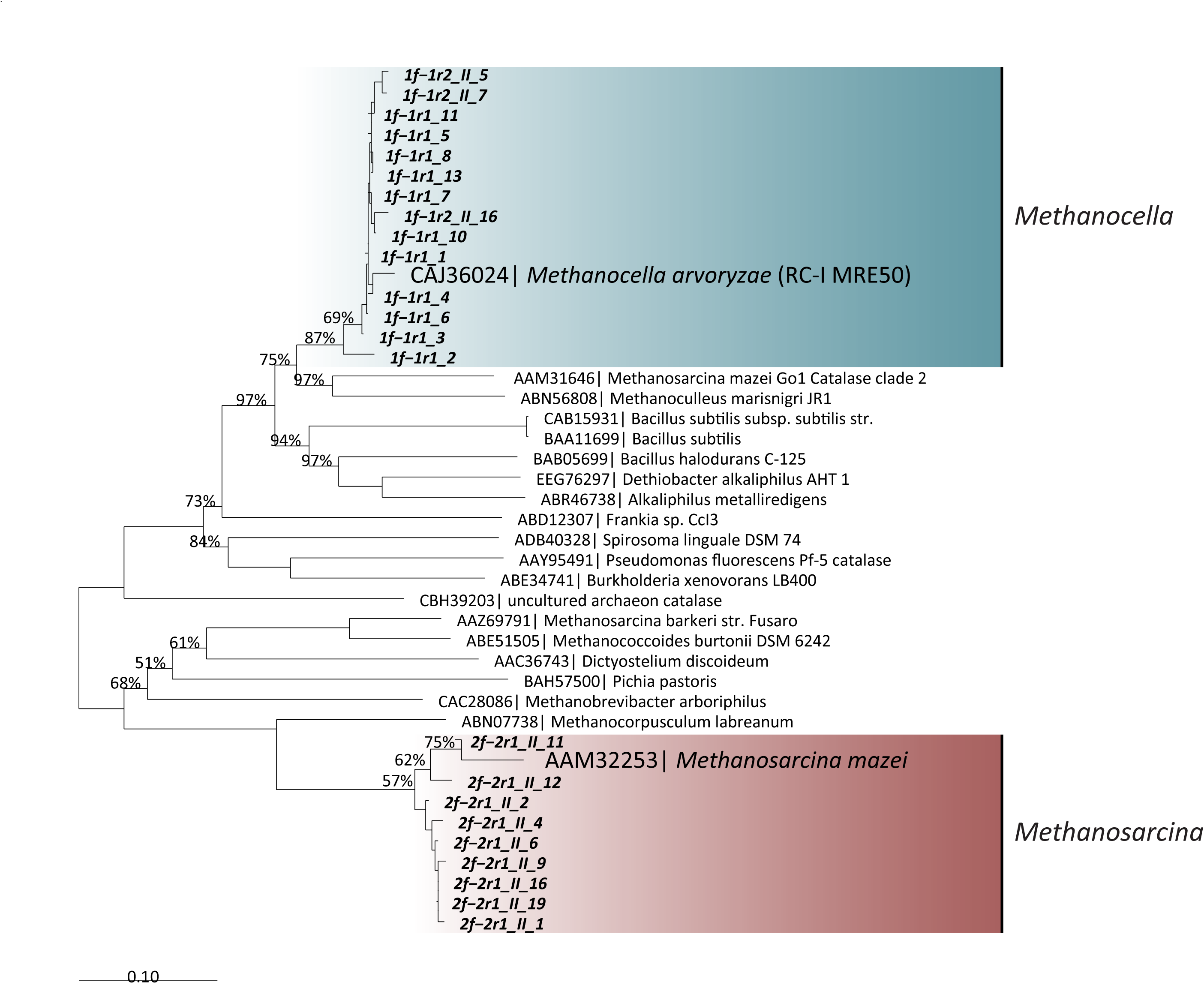

Supplement: Figure S4 — Maximum likelihood phylogenetic tree based on aligned partial amino acid sequences of the catalase E gene ( katE ). Sequences were obtained using katRCI and katMsr primer pairs targeting the katE of Methanocella and Methanosarcina, respectively. Amino acid composition was deduced from DNA sequences and aligned against an ARB database of catalase sequences. The tree was calculated with RAxML 7.04 using rapid hill climbing algorithm and PROTMIX-JTT evolutionary model. Bootstrap values above 50% (out of a 100 trials) are displayed next to the nodes. (TIF) [file pone.0020453.s004.tif]
