# Supplementary material for: Activation of Methanogenesis in Arid Biological Soil Crusts Despite the Presence of Oxygen
Source: PLoS One. 2011 May 31;6(5):e20453. doi: 10.1371/journal.pone.0020453 (PMC3105065; doi:10.1371/journal.pone.0020453)
Supplement: Materials and Methods S1 — (DOC) [file pone.0020453.s005.doc]

Supplementary Materials and Methods

**Nucleic acids extraction.** Total nucleic acids were extracted the following way: 0.5 g of frozen soil sample was placed in a Lysing Matrix E tube (MP). 375 µl SPB (10 mM sodium phosphate buffer, pH 8.0) and 125 µl TNS buffer (500 mM Tris HCL, 100 mM NaCl, 10% sodium dodecyl sulphate) were added to the tube, both solutions taken from Henckel and colleagues[1]. Then, 500 µl of Tris EDTA saturated phenol solution (pH 8.0; Fluka) were added to the tube and it was processed immediately in a FastPrep®-24 bead beating instrument for 30 s at 6.5 m s^-1^. This process was repeated twice more with fresh aliquots of buffers and phenol solution. Following phenol/chloroform/isoamylalcohol (25:24:1; Fluka) and chloroform/ isoamylalcohol (24:1; Fluka) purifications, total nucleic acids were precipitated using 1 µl of glycogen (5 mg ml^-1^; Ambion) and 1.5 ml PEG precipitation solution (20% polyethylen glycol, 2.5 M NaCl), washed once with 75% cold ethanol and resuspended in 200 µl of low TE buffer (10 mM Tris-HCl, 0.1 mM EDTA). Total nucleic acids were digested with TURBO™ DNase (Ambion) and later purified using RNeasy® MinElute® Cleanup Kit (Qiagen). Complete DNA removal was verified by failure to obtain PCR amplification product with the purified RNA template using the conditions described below. cDNA was synthesized from 500 ng of purified RNA using SuperScript™ III reverse transcriptase (Invitrogen) according to the manufacturer’s instructions. Random hexamer primers (0.5 µg) were used for complete cDNA synthesis which was used for amplification of the 16S rRNA and catalase (*KatE*) genes, while for amplification of the mcrA gene 2 pmol of the mcrA-rev primer (Table S3) were used for *mcrA* cDNA synthesis.

**Molecular characterization of archaeal 16S rRNA, *mcrA* and *KatE* genes.** Table S4 summarizes the primers and probes used in this study to monitor and quantify the Archaeal population. For reconstruction of phylogenetic trees we used the ARB software package[2]. Analysis and oligonucleotide design of 16S rRNA gene sequences were done using the Silva database[3], while for analyses of *mcrA* and *KatE* gene sequences specific ARB databases were built. The *mcrA* database was built from 5200 translated nucleic acid sequences and aligned using Promals[4]. Catalase database was based on 26 translated nucleic acid sequences and aligned in ARB using the Clustal algorithm[5]. All sequences were obtained from EMBL (http://www.ebi.ac.uk/embl/) and the databases are available at: http://www.staff.uni-marburg.de/~angel/. Reconstruction of the catalase gene maximum likelihood phylogenetic tree was conducted with RAxML 7.04 using rapid hill climbing algorithm and PROTMIX-JTT evolutionary model[6]. The new qPCR probe and PCR primers used in this study were designed using PRIMROSE[7]. Primers for *katE* of *Methanocella* and *Methanosarcina* were designed from the catalase gene sequences of *Methanocella arvoryzae* (formerly RC-I_MRE50_; CAJ36024) and *Methanosarcina mazei* (AAM32253), respectively. qPCR probe for *mcrA* of *Methanocella* was designed from the environmental sequences obtained in this study as well as from the *mcrA* gene sequence of *Methanocella arvoryzae* (formerly RC-I_MRE50_ ; YP_686530.1).

For sequencing of *mcrA* the primer pair mlas-mod – mcrA-rev was used and for *katE* the primer pairs katRCI and katMsr were used. As templates for both clone libraries we used DNA obtained from FDN and FLO treatments. Libraries were constructed using purified PCR products and pGEM-T Easy cloning kit (Promega). PCR amplifications were done using the following mixture: Each PCR reaction contained 5 µl 10X AccuPrime™ PCR Buffer II (Invitrogen), additional 1.5 mM MgCl_2_ (to a final concentration of 3 mM), 0.5 µM of each primer (Sigma), 50 µg BSA (Roche), 1 µl of Taq DNA polymerase (Invitrogen) and 2 µl of template. The following program was used for *katE* amplification: 94 °C for 4 min followed by 10 touchdown cycles of 94 °C for 1 min, 62 °C - 1°C for 1 min and 68 °C for 1 min followed by 25 cycles of 94 °C for 1 min, 52 °C for 1 min and 68 °C for 1 min, and a single step of final elongation at 68 °C for 10 min. For amplification of the *mcrA* gene the following program was used: 94 °C for 4 min followed by 5 touchdown cycles of 94 °C for 30 s, 60 °C – 1 °C for 45 s and 72 °C for 30 s followed by 30 cycles of 94 °C for 30 s, 55 °C for 30 s and 72 °C for 30 s, and a single step of final elongation at 72 °C for 10 min. Sanger sequencing services were provided by GATC (Germany). The primer M13r targeting flanking regions of the insert was used to sequence a total of 96 clones (48 from each library) of *mcrA* and 48 clones of *katE* (12 from each library and primer pair). Verification for lack of transcription of *pmmo* was done using RT-PCR as described previously[8].

**Gene and transcript quantification using qPCR.** All qPCR reactions were performed on an iCycler thermocycler equipped with a MyiQ™ detection system and the data was analyzed using iQ5 Optical System software (Bio-Rad). Quantification of universal archaeal 16S rRNA gene, *mcrA* and *katE* copy numbers were based on SYBR® Green. Each reaction was 25 µl in volume and contained the following mixture: 12.5 µl SYBR® Green JumpStart™ Taq ReadyMix™, 3 mM MgCl_2_, 0.8 µg µl^-1^ BSA (Ambion), 0.66 (16S rRNA gene) or 0.5 (other) µM of each primer, and 5 or 2 µl of template (DNA and cDNA resp.). The program used was: 94°C for 5 min followed by 40 cycles of 94°C for 30 s, 66 (16S rRNA gene) or 57°C (other) for 45 s, 72°C for 30 s and 84°C for 10 s for signal reading. Relative quantification of *katE* was performed using the 2^-ΔΔCT^ method, as previously described[9]

The essays targeting the 16S rRNA and *mcrA* genes of *Methanosarcina* and *Methanocella* were based on dual labelled probes. Each reaction was 25 µl in volume and contained the following mixture: 12.5 µl JumpStart™ Taq ReadyMix™, 4 mM MgCl_2_, 0.8 µg µl^-1^ BSA (Ambion), 0.5 µM of each primer, 0.2 µM of the dual labelled probe and 5 or 2 µl of template (DNA and cDNA resp.). For the 16S rRNA gene essays, the program used was: 94°C for 5 min followed by 40 cycles of 94°C for 30 s, 57°C (*mcrA* gene) or 60°C (16S rRNA gene) for 45 s for annealing and 62°C for 30 s for elongation and signal reading. DNA standard for the universal archaeal and *Methanosarcina* 16S rRNA gene essays was prepared from a pure culture of *Mehtanosarcina thermophila* according to Lueders and colleagues[10], while for *Methanocella* 16S rRNA gene essay it was prepared from a clone containing 16S rRNA gene affiliated with *Methanocella* as a plasmid insert. A standard for the universal *mcrA* essay and the *Methanosarcina* *mcrA* essays was prepared from a pure culture of *Mehtanosarcina thermophila*. For *Methanocella* *mcrA* essay, a clone containing an environmental sequence was used as template for a standard. For quantification of cDNA, mRNA transcripts of *mcrA* were synthesized from the clones used for making the DNA standard using Riboprobe® (Promega). Remaining DNA was digested using TURBO™ DNase (Ambion) and the RNA was purified using RNeasy® MinElute® Cleanup Kit (Qiagen). Pure RNA was quantified using Quant-iT™ RiboGreen® (Invitrogen) and cDNA was synthesized using mcrA-rev primer. The cDNAs were used as standards for quantification of *mcrA* transcripts.

References

1. Henckel T, Friedrich M, Conrad R (1999) Molecular analyses of the methane-oxidizing microbial community in rice field soil by targeting the genes of the 16S rRNA, particulate methane monooxygenase, and methanol dehydrogenase. Appl Environ Microbiol 65: 1980-1990.

2. Ludwig W, Strunk O, Westram R, Richter L, Meier H, et al. (2004) ARB: a software environment for sequence data. Nucl Acids Res 32: 1363-1371.

3. Pruesse E, Quast C, Knittel K, Fuchs BM, Ludwig W, et al. (2007) SILVA: a comprehensive online resource for quality checked and aligned ribosomal RNA sequence data compatible with ARB. Nucl Acids Res 35: 7188-7196.

4. Pei J, Grishin NV (2007) PROMALS: towards accurate multiple sequence alignments of distantly related proteins. Bioinformatics 23: 802-808.

5. Higgins DG, Sharp PM (1988) CLUSTAL: a package for performing multiple sequence alignment on a microcomputer. Gene 73: 237-244.

6. Stamatakis A (2006) RAxML-Vi-Hpc: maximum likelihood-based phylogenetic analyses with thousands of taxa and mixed models. Bioinformatics 22: 2688-2690.

7. Ashelford KE, Weightman AJ, Fry JC (2002) PRIMROSE: a computer program for generating and estimating the phylogenetic range of 16S rRNA oligonucleotide probes and primers in conjunction with the RDP-II database. Nucl Acids Res 30: 3481-3489.

8. Angel R, Conrad R (2009) *in situ* measurement of methane fluxes and analysis of transcribed particulate methane monooxygenase in desert soils. Environ Microbiol 11: 2598-2610.

9. Livak KJ, Schmittgen TD (2001) analysis of relative gene expression data using real-time quantitative PCR and the 2^-ΔΔC^T method. Methods 25: 402-408.

10. Lueders T, Manefield M, Friedrich MW (2004) Enhanced sensitivity of DNA- and rRNA-based stable isotope probing by fractionation and quantitative analysis of isopycnic centrifugation gradients. Environ Microbiol 6: 73-78.
