# Supplementary material for: Activation of Methanogenesis in Arid Biological Soil Crusts Despite the Presence of Oxygen
Source: PLoS One. 2011 May 31;6(5):e20453. doi: 10.1371/journal.pone.0020453 (PMC3105065; doi:10.1371/journal.pone.0020453)
Supplement: Table S1 — ANOVA analyses (least squares) testing the effect of the various incubation conditions on methane production rates (nmol d−1 gdw−1; days 14–42) and ratios of gene and transcript copies. (DOC) [file pone.0020453.s006.doc]

**Supplementary Table 1. ANOVA analyses (least squares) testing the effect of the various incubation conditions on methane production rates (nmol d^-1^ gdw^-1^**; days 14 – 42) and ratios of gene and transcript copies

|  | Log methane production rate | | | 16S MSL/ARC* | | | msar/mcrA gen. DNA** | | | msar/mcrA gen. cDNA† | | |
| --- | --- | --- | --- | --- | --- | --- | --- | --- | --- | --- | --- | --- |
| Source of variation | Mean square | F | Sig. ††  P > |F| | Mean square | F | Sig.  P > |F| | Mean square | F | Sig.  P > |F| | Mean square | F | Sig.  P > |F| |
| Light | 6.45 | 59.61 | <0.01 | 0.64 | 12.74 | <0.01 | 0.80 | 8.70 | 0.01 | 0.36 | 8.25 | 0.01 |
| Oxygen | 5.82 | 53.82 | <0.01 | 0.44 | 8.62 | 0.01 | 0.84 | 9.11 | 0.01 | 0.45 | 10.16 | 0.01 |
| Flooding | 1.32 | 12.25 | 0.03 | 0.02 | 0.39 | 0.54 | 0.00 | 0.00 | 0.99 | 0.13 | 2.98 | 0.10 |
| Light * Oxygen | 10.14 | 93.75 | <0.01 | 0.15 | 3.02 | 0.10 | 0.00 | 0.00 | 0.96 | 0.84 | 19.20 | <0.01 |
| Light * Flooding | 0.43 | 3.99 | 0.06 | 0.08 | 1.59 | 0.22 | 0.70 | 7.57 | 0.01 | 0.00 | 0.04 | 0.85 |
| Oxygen * Flooding | 0.02 | 0.20 | 0.66 | 0.01 | 0.15 | 0.70 | 0.15 | 1.63 | 0.22 | 0.53 | 12.04 | <0.01 |
| Light * Oxygen * Water | 0.29 | 2.76 | 0.12 | 0.11 | 2.24 | 0.15 | 0.08 | 0.85 | 0.37 | 0.05 | 1.10 | 0.31 |
| Error§ | 0.11 |  |  | 0.05 |  |  | 0.09 |  |  | 0.04 |  |  |

* Ratio of 16S rRNA gene copies of *Methanosarcina* to general Archaea gene copies

** Ratio of *mcrA* gene copies of *Methanosarcina* to general *mcrA* gene copies

† Ratio of *mcrA* transcript copies of *Methanosarcina* to general *mcrA* transcript copies

†† Values in red are significant at P < 0.05

§ Degrees of freedom for error in all tests: 16
