# Supplementary material for: Activation of Methanogenesis in Arid Biological Soil Crusts Despite the Presence of Oxygen
Source: PLoS One. 2011 May 31;6(5):e20453. doi: 10.1371/journal.pone.0020453 (PMC3105065; doi:10.1371/journal.pone.0020453)
Supplement: Table S2 — Pearson correlation coefficients (r) between various measured variables*. (DOC) [file pone.0020453.s007.doc]

**Supplementary Table 2. Pearson correlation coefficients (r) between various measured variables***

|  | *mcrA general DNA* | *mcrA general cDNA* | *mcrA mcell DNA* | *mcrA mcell cDNA* | *mcrA msar DNA* | *mcrA msar cDNA* | *16S Arc*  *DNA* | *16S MCL*  *DNA* | *16S MSL*  *DNA* | *CH_4_ production rate* | *Anoxic boundary*  *(depth)* | *Water content* | *16S MCL /16S tot* | *16S MSL /16S tot* |
| --- | --- | --- | --- | --- | --- | --- | --- | --- | --- | --- | --- | --- | --- | --- |
| mcrA general DNA | 0.96 | ** |  |  |  |  |  |  |  |  |  |  |  |  |
| mcrA mcell DNA | 0.93 | 0.84 |  |  |  |  |  |  |  |  |  |  |  |  |
| mcrA mcell cDNA | 0.71 | 0.62 | 0.86 |  |  |  |  |  |  |  |  |  |  |  |
| mcrA msar DNA | 0.66 | 0.68 | 0.48 | 0.39 |  |  |  |  |  |  |  |  |  |  |
| mcrA msar cDNA | 0.50 | 0.58 | 0.34 | 0.20 | 0.92 |  |  |  |  |  |  |  |  |  |
| 16S Arc DNA | 0.94 | 0.90 | 0.83 | 0.66 | 0.71 | 0.59 |  |  |  |  |  |  |  |  |
| 16S MCL DNA | 0.92 | 0.79 | 0.89 | 0.75 | 0.56 | 0.30 | 0.86 |  |  |  |  |  |  |  |
| 16S MSL DNA | 0.93 | 0.92 | 0.81 | 0.69 | 0.85 | 0.69 | 0.95 | 0.86 |  |  |  |  |  |  |
| CH_4_ production rate | 0.95 | 0.92 | 0.89 | 0.69 | 0.77 | 0.62 | 0.86 | 0.87 | 0.93 |  |  |  |  |  |
| Anoxic boundary | -0.89 | -0.89 | -0.85 | -0.59 | -0.69 | -0.64 | -0.79 | -0.74 | -0.83 | -0.96 |  |  |  |  |
| Water content | 0.45 | 0.26 | 0.31 | 0.05 | 0.27 | 0.06 | 0.45 | 0.65 | 0.38 | 0.39 | -0.26 |  |  |  |
| 16S MCL/16S tot | 0.52 | 0.31 | 0.62 | 0.59 | 0.13 | -0.20 | 0.33 | 0.77 | 0.38 | 0.51 | -0.36 | 0.62 |  |  |
| 16S MSL/16S tot | 0.94 | 0.92 | 0.82 | 0.69 | 0.86 | 0.69 | 0.93 | 0.87 | 1.00 | 0.95 | -0.86 | 0.39 | 0.41 |  |
| 16S MSL/16S MCL | 0.82 | 0.88 | 0.67 | 0.59 | 0.89 | 0.81 | 0.89 | 0.66 | 0.95 | 0.82 | -0.75 | 0.15 | 0.10 | 0.93 |

* Parameters are coded as follows: mcrA - *mcrA* gene copies; 16S - 16S rRNA gene copies; general - total number; mcell or MCL - *Methanocella*; msar or MSL – *Methanosarcina*

** Values in red represent strong correlation (> 0.75)
