# Supplementary material for: Activation of Methanogenesis in Arid Biological Soil Crusts Despite the Presence of Oxygen
Source: PLoS One. 2011 May 31;6(5):e20453. doi: 10.1371/journal.pone.0020453 (PMC3105065; doi:10.1371/journal.pone.0020453)
Supplement: Table S3 — Major fermentation products (µM) in the pore water of the microcosms. (DOC) [file pone.0020453.s008.doc]

**Supplementary Table 3. Major fermentation products (µM) in the pore water of the microcosms. Means ± 1 SE.**

| *Treatment** | *Malate* | *Succinate* | *Lactate* | *Formate* | *Acetate* | *Propionate* | *Butyrate* |
| --- | --- | --- | --- | --- | --- | --- | --- |
| F L N | 113.7 ± 16.2 | 2.7 ± 2.7 | 8.7 ± 8.7 | 9.2 ± 9.2 | 76.9 ± 42.8 | BLD | 83.5 ± 34.5 |
| F L O | 60.3 ± 9.2 | BLD | 6.5 ± 6.5 | BLD | 8.9 ± 8.9 | BLD | 67.5 ± 30.5 |
| W L N | 157.6 | BLD | BLD | BLD | BLD | BLD | 167.0 |
| W L O | 131.2 ± 10.0 | 7.8 ± 6.7 | 16.4 ± 16.4 | BLD | BLD | 5.2 ± 5.2 | 110.3 ± 101.5 |
| F D N | 49.0 ± 5.1 | 43.6 ± 5.3 | 4.7 ± 4.7 | 84.7 ± 52.2 | 303.8 ± 286.1 | 685.1 ± 685.1 | 293.4 ± 293.4 |
| F D O | 46.1 ± 5.5 | 42.8 ± 2.8 | BLD | 18.9 ± 12.9 | 69.8 ± 19.3 | BLD | 9.2 ± 9.2 |
| W D N | 58.7 ± 6.2 | 28.9 ± 4.0 | 3.7 ± 3.7 | BLD | 12205.8 ± 1181.3 | 5168.9 ± 631.8 | 1975.0 ± 954.5 |
| W D O | 59.2 ± 34.2 | 31.1 ± 7.7 | 14.3 ± 7.4 | BLD | 49.5 ± 20.56 | 7.9 ± 4.6 | 17.9 ± 15.7 |

*Treatment codes are as follows: flooded – F, drained – D, light – L, dark – d, N_2_ atm. – N, 21% O_2_ atm. – O.

BLD: below limits of detection.
