# Supplementary material for: Activation of Methanogenesis in Arid Biological Soil Crusts Despite the Presence of Oxygen
Source: PLoS One. 2011 May 31;6(5):e20453. doi: 10.1371/journal.pone.0020453 (PMC3105065; doi:10.1371/journal.pone.0020453)
Supplement: Table S4 — Primers and Probes used in this study. (DOC) [file pone.0020453.s009.doc]

Supplementary Table 4. Primers and Probes used in this study

| *Oligo. name** | | *Target* | | *Oligo. sequence (5’-3’)* | | *Position*** | | *GC (%)* | *Tm†* | *Amplicon size* | *Essay* | *Reference* |
| --- | --- | --- | --- | --- | --- | --- | --- | --- | --- | --- | --- | --- |
|  | ARC364 - F | | Archaea 16S rRNA gene | | CGGGGYGCASCAGGGGCGAA | | 364-383 | 75-80 | 75 | 553 | QPCR | 12 |
|  | ARCH 934 - R | |  |  | GTGCTCCCCCGCCAATTCCT | | 915-934 | 65 | 71 |  |  | 13 |
|  | MSL812 - F | | Methanosarcinales  16S rRNA gene | | GTAAACGATRYTCGCTAGGT | | 812-831 | 40-50 | 62 | 354 |  |  |
|  | MSL860 - P | |  |  | AGGGAAGCCGTGAAGCGARCC | | 860-880 | 62-67 | 70 |  | QPCR | 14 |
|  | MSL1159 - R | |  |  | GGTCCCCACAGWGTACC | | 1143-1159 | 65 | 63 |  |  |  |
|  | MCL282 - F | | *Methanocella*/ RC-I  16S rRNA gene | | ATCMGTACGGGTTGTGGG | | 282-299 | 56-61 | 65 | 510 |  |  |
|  | MCL609 - P | |  |  | ATCCAGCGGCTTAACCGTTGGKCK | | 609-632 | 54-63 | 72 |  | QPCR | This study |
|  | MCL832 - R | |  |  | CACCTAGCGRGCATCGTTTAC | | 813-832 | 52-57 | 64 |  |  |  |
|  | mlas-mod - F | | Universal *mcrA* gene | | GGYGGTGTMGGDTTCACMCARTA | | 976-998 | 43-65 | 68 | 469 | PCR/ QPCR | Modified from: ^15^ |
|  | mcrA-rev - R | |  |  | CGTTCATBGCGTAGTTVGGRTAGT | | 1421-1444 | 42-54 | 66 |  |  | 15 |
|  | msar - P | | *Methanosarcina* *mcrA* gene | | TCTCTCWGGCTGGTAYCTCTCCATGTAC | | 1269-1296 | 50-54 | 68 |  | QPCR | 15 |
|  | mcel/rc-I - P | | *Methanocella* *mcrA* gene | | CTVGGMTTCTWCGGBTACGACYT | | 1269-1296 | 43-61 | 66 |  | QPCR | This study |

Supplementary Table 4. Continued

| *Oligo. name** | *Target* | *Oligo. sequence (5’-3’)* | | *Position*** | | *GC (%)* | | *Tm†* | | *Amplicon size* | | *Essay* | | *Reference* | |
| --- | --- | --- | --- | --- | --- | --- | --- | --- | --- | --- | --- | --- | --- | --- | --- |
| katMcl - F | *Methanocella arvoryzae* *KatE* gene | AAGCCACACACATAGACGTC | 1321-1343 | | 50 | | 63 | | 586 | | PCR/ QPCR | | This study | |  |
| KatMcl - R |  | CATGATCATGTGGGCGTTCT | 40-626 | | 50 | | 64 | |  |  |  |  |  |  |  |
| katMsr - F | *Methanosarcina* *mazei* *KatE* gene | CAATTAGGTGAGCCGCTTCC | 20-39 | | 55 | | 64 | | 605 | | PCR/ QPCR | | This study | |  |
| katMsr - R |  | CCGCGGTCACCCATAATAAT | 605-624 | | 50 | | 63 | |  | |  | |  | |  |

* The following primer name suffixes are used: - F – forward primer, - R – reverse primer, - P – dual labelled probe.

** Position is based on the following: primers targeting the 16S rRNA gene - *E.coli*; primers targeting the mcrA gene - *Methanothermobacter* *thermautotrophicus* mcrA gene accession number: AAA73445 (following Steinberg and Regan, 2009); KatRCI primers - *Methanocella* putative catalase gene (*KatE*), accession number: CAJ36024; KatMsl primers - *Methanosarcina mazei* *KatE* gene, accession number: AAM32253.

† Calculated using Nearest Neighbor method with OligoAnalyzer 3.1 (http://eu.idtdna.com/analyzer/Applications/OligoAnalyzer/) under the conditions described for each PCR/QPCR reaction in the Experimental procedure section
